# The risk trajectory of different cardiovascular morbidities associated with chronic kidney disease among patients with newly diagnosed diabetes mellitus: a propensity score-matched cohort analysis

**DOI:** 10.1186/s12933-021-01279-6

**Published:** 2021-04-24

**Authors:** Chia-Ter Chao, Szu-Ying Lee, Jui Wang, Kuo-Liong Chien, Kuan-Yu Hung

**Affiliations:** 1 Neprology Division, Department of Internal Medicine, National Taiwan University Hospital BeiHu Branch, Taipei, Taiwan; 2 Geriatric and Community Medicine Research Center, National Taiwan University Hospital BeiHu Branch, Taipei, Taiwan; 3 Graduate Institute of Toxicology, National Taiwan University College of Medicine, Taipei, Taiwan; 4 Nephrology Division, Department of Internal Medicine, National Taiwan University Hospital Yunlin Branch, Yunlin County, Taiwan; 5 Institute of Epidemiology and Preventive Medicine, College of Public Health, National Taiwan University, Taipei, Taiwan; 6 Nephrology Division, Department of Internal Medicine, National Taiwan University Hospital, Taipei, Taiwan

**Keywords:** Cardiovascular disease, Chronic kidney disease, Diabetes kidney disease, Diabetes mellitus, Microvascular complication, Myocardial infarction, Peripheral vascular disease, Stroke

## Abstract

**Background:**

Chronic kidney disease (CKD) introduces an increased cardiovascular risk among patients with diabetes mellitus (DM). The risk and tempo of cardiovascular diseases may differ depending upon their type. Whether CKD differentially influences the risk of developing each cardiovascular morbidity in patients with newly diagnosed DM remains unexplored.

**Methods:**

We identified patients with incident DM from the Longitudinal Cohort of Diabetes Patients (LCDP) cohort (n = 429,616), and uncovered those developing CKD after DM and their propensity score-matched counterparts without. After follow-up, we examined the cardiovascular morbidity-free rates of patients with and without CKD after DM, followed by Cox proportional hazard regression analyses. We further evaluated the cumulative risk of developing each outcome consecutively during the study period.

**Results:**

From LCDP, we identified 55,961 diabetic patients with CKD and matched controls without CKD. After 4.2 years, patients with incident DM and CKD afterward had a significantly higher risk of mortality (hazard ratio [HR] 1.1, 95% confidence interval [CI] 1.06–1.14), heart failure (HF) (HR 1.282, 95% CI 1.19–1.38), acute myocardial infarction (AMI) (HR 1.16, 95% CI 1.04–1.3), and peripheral vascular disease (PVD) (HR 1.277, 95% CI 1.08–1.52) compared to those without CKD. The CKD-associated risk of mortality, HF and AMI became significant soon after DM occurred and remained significant throughout follow-up, while the risk of PVD conferred by CKD did not emerge until 4 years later. The CKD-associated risk of ischemic, hemorrhagic stroke and atrial fibrillation remained insignificant.

**Conclusions:**

The cardiovascular risk profile among incident DM patients differs depending on disease type. These findings can facilitate the selection of an optimal strategy for early cardiovascular care for newly diagnosed diabetic patients.

**Supplementary Information:**

The online version contains supplementary material available at 10.1186/s12933-021-01279-6.

## Introduction

The burden of diabetes mellitus (DM) becomes increasingly heavy worldwide. According to the 2019 Diabetes Atlas, more than 100 million older adults had DM, accounting for nearly one-fifth of global population within this age stratum [1]. It is projected that over 4 million adults with DM will die from diabetes and related complications annually [2], and global healthcare expenditure for DM continues to grow over time [3]. Nearly 53% of healthcare costs related to diabetes are spent in complication detection and treatment [4]. To optimize early complication care, it is important to gain more understanding on the types of diabetic complications and their risk trajectories over time.

Cardiovascular diseases are the most renowned complications and the most common causes of mortality in patients with DM. Secondary only to diabetic nephropathy, cardiovascular diseases directly and indirectly contribute to the soaring healthcare costs of DM [5]. Findings from the Framingham Heart Study showed that DM increased the risk of cardiovascular diseases by two to threefold [6]. Although a consistent trend of decreasing cardiovascular complication prevalence has been demonstrated, the overall burden remains a formidable challenge judging from the rising DM incidence [7]. Guidelines for optimal care of patients with DM now place much emphasis on the importance of complication detection and management in combination with comorbidity care [8], especially that for cardiovascular diseases.

Chronic kidney disease (CKD), also a major diabetic microvascular complication (termed diabetic kidney disease [DKD] based on defined clinical criteria [9]), constitutes another public health concern. Survey results from the Global Burden of Disease report showed that the age-standardized prevalence of DKD was 15 to 16 per 1000 [10]. A recent systematic review revealed that 31.3% patients with incident end-stage renal disease (ESRD) were due to diabetic nephropathy, and the annual incidence of ESRD among diabetic patients increased approximately threefold over 2 decades [11]. Approximately 20% to 40% of patients with DM have DKD, which confers a high risk of cardiovascular events. However, the risk and tempo of cardiovascular disease development differed depending upon the type of cardiovascular diseases of interest [12]; Shah et al. [13], using a large population-based registry, demonstrated that heart failure (HF) and peripheral vascular disease (PVD) were the most common initial cardiovascular presentations among patients with DM within 5.5 years of follow-up, while the risk of arrhythmia and sudden cardiac death was insignificant during follow-up. Moreover, the types of cardiovascular diseases that occur after the diagnosis of DM also vary with regard to their influences on patient survival [14]. Whether the presence of CKD exerts differential influences on the incidence of different cardiovascular morbidities in patients with newly diagnosed DM remains unexplored, and the temporal trend of the risk associated with cardiovascular morbidities likely affects the timing of treatment administration. In the current study, we harnessed a well-established and maintained cohort of diabetic patients to examine their risk trajectory of developing a wide spectrum of cardiovascular complications.

## Subjects, materials and methods

### Recruitment of study participants and the follow up procedure

We harnessed the Longitudinal Cohort of Diabetes Patients (LCDP) cohort, which was assembled based on a random annual sampling of patients from all administrative regions covered by the Taiwan National Health Insurance with at least one time of DM diagnosis, regardless of type 1 or type 2, from the National Health Insurance Research Database (NHIRD), between 2004 and 2010. Patients from the LCDP cohort are representative of the entire population in Taiwan, and epidemiological findings based on analyses of this cohort are shown to enrich the understanding of diabetic care [15–17]. Since incident and prevalent cases of DM were admixed within the LCDP cohort, we further imposed selection criteria to identify those with incident DM. We specifically excluded those with any DM diagnosis prior to 2004, the first year of LCDP database establishment, and screened for those with ≥ 3 times of outpatient or ≥ 1 time of inpatient DM diagnosis to ascertain the presence of incident DM during the study period [18]. Exclusion criteria consisted of patients younger or equal to 20-year-old; those with missing data (gender); those who missed follow-up; those who developed the pre-specified outcomes, comprising of mortality and the cardiovascular morbidities prior to the index date; and those with an inadequate length of follow-up (< 1 year) in order to allow for sufficient incidence of outcomes to be observable.

All identifying information with the linked reimbursement data were anonymized prior to cohort assembly. We collected participants’ demographic profiles (age and gender), lifestyle factors (smoking and alcoholism), the year of DM diagnosis being ascertained, comorbidities (hypertension, hyperlipidemia, obesity, gout, hepatic, pulmonary, neurological disorders, and any malignancy), medications with influences on the risk of developing cardiovascular events, and anti-diabetic medications use. The complete list of diagnostic codes for identifying each comorbidity has been published in our prior work [15, 19].

After applying the exclusion criteria, we divided the enrolled participants into those with and without CKD, according to our prior work [16, 17, 20]. In brief, the diagnosis of CKD was made based on the presence of ≥ 3 times of outpatient or ≥ 1 time of inpatient diagnosis (016.0, 042, 095.4, 189, 223, 236.9, 250.4, 271.4, 274.1, 403–404, 440.1, 442.1, 446.21, 447.3, 572.4, 580–589, 590–591, 593, 642.1, 646.2, 753, and 984), as adopted by others in the literature [21, 22]. These diagnostic code combinations intend to capture “kidney damages” that persist for months with or without functional measures, a concept that encompasses a wider range of structural or functional kidney disorders and is compliant with the definition proposed by the recent Kidney Disease Improving Global Outcomes (KDIGO) consensus [23]. A prior study evaluated the validity of CKD code combinations in Taiwan NHIRD; Wu et al. examined the risk of coronary events conferred by having a baseline diagnosis of acute kidney injury, accounted for the influences of CKD using NHIRD data [24]. In that study, they verified the CKD code combinations also used in our study, based on validation results from a multicenter cohort [24]. They showed that the sensitivity and specificity of these codes to diagnose CKD were 81.8% and 99.3%, respectively, with positive and negative predictive values of 87.3% and 99.0%, respectively. After cases of CKD recognized, we performed propensity score-matching, in which we selected those with CKD and matched them to another group of diabetic patients without CKD at a 1:1 ratio, using collected variables (demographic variables, lifestyle factors, the year of DM diagnosis, comorbidities, and relevant medications).

After selecting patients with CKD after incident DM and their matched patients without CKD, we followed up them until death, the development of any pre-determined cardiovascular morbidities, or the end of this study (December 31st, 2011).

### Study outcomes

In this study, we recognized the following events as the study outcomes, including mortality and cardiovascular morbidities (HF, acute myocardial infarction [AMI], PVD, ischemic stroke [IS], hemorrhagic stroke [HS], and atrial fibrillation [Afib]). The diagnosis and procedure codes for detecting these cardiovascular morbidities have been detailed in the Additional file 1: Table S1. The validity of these diagnostic codes has been reassured previously [15, 25]. For example, the validity of codes used to diagnose DM [26], IS [27], and other morbidities [28] in Taiwan NHIRD have been tested previously using different cohorts, with fair results. The positive predictive value of using these codes to identify IS events in NHIRD was found to be between 90 and 95% [27]. The comorbidity code combinations we used in this study generally conformed to those used in the above studies. The development of each event was confirmed if these diagnostic codes occurred during follow-up.

### Statistical analysis

For continuous and categorical variables, we used means ± standard deviations and numbers with percentages in parentheses, respectively, for description. We compared continuous and categorical variables between matched participants with and without CKD after DM using the Student’s *t*-test and chi-square test, respectively. The distribution of each variable between the CKD patients and their propensity score-matched controls was assessed by the standardized mean differences (SMDs). After follow-up, the Kaplan–Meier technique was utilized to examine the survival and event-free curves of incident diabetic patients with and without CKD after DM, followed by comparisons with the log-rank test. We subsequently performed Cox proportional hazard regression analyses with mortality or the development of each cardiovascular morbidity as the dependent variable, accounting for demographic profile, lifestyle factors, the year of DM diagnosis, comorbidities, medications (including anti-diabetic ones), and having CKD after DM or not. Sensitivity analyses were carried out, using two approaches: first, a restrictive set of diagnostic codes was selected to identify CKD (250.4, 271.4, 274.1, 403–404, 440.1, 442.1, 446.21, 447.3, 572.4, 580–589, 590–591, 593, and 642.1) with greater specificity, followed by propensity score-matching and Cox proportional hazard regression analyses. Alternatively, a DKD-specific set of diagnostic codes (250.4×, 271.4, 581.8, and 581.9) was used to identify a subcohort, followed by the same analytic processes.

We further evaluated the risk of developing each outcome according to the year after DM diagnosis during the study period, using multiple regression analyses accounting for the same set of variables similar to those analyzed in the Cox regression models. We plotted the odds ratios (ORs) with 95% confidence intervals (CIs) of each outcome over time to observe whether the respective risk associated with CKD altered following incident DM in the original and the sensitivity analyses. All statistical analyses were conducted by the SAS software (SAS institute, NC, USA). A two-tailed *p* < 0.05 was considered statistically significant.

### Ethical statement

The current study has been approved as part of the project by the institutional review board of National Taiwan University Hospital (NO. 201802063W). As described above, participants’ identifying information has been delinked prior to database establishment; therefore, informed consent was considered unnecessary and waived for the current study. The study protocol adhered to the Declaration of Helsinki.

## Results

**Fig. 1:**
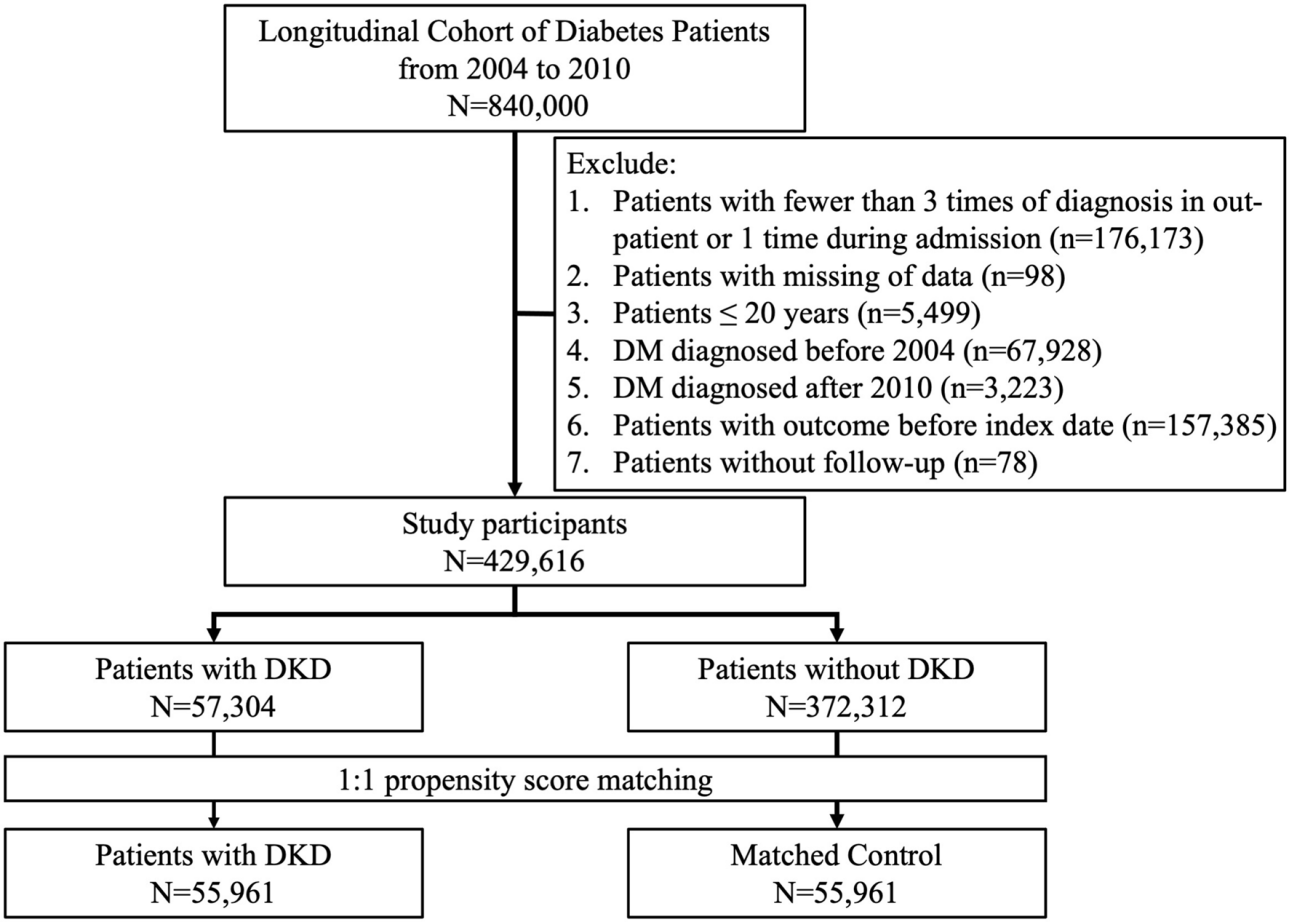
The participant selection algorithm of this study. *DKD* diabetic kidney disease, *DM* diabetes mellitus

A total of 840,000 patients with DM from the LCDP cohort entered the selection process. We further identified adult cases with incident DM and an adequate length of follow-up but without any of the cardiovascular morbidities, by applying the exclusion criteria to these patients (Fig. 1). Among all, 51.1% (n = 429,616) remained after selection, with 13.3% (n = 57,304) having CKD. From these patients with CKD after DM, we identified 55,961 patients and propensity score-matched them to 55,961 without CKD (Fig. 1).

There were no significance differences between incident diabetic patients with and without CKD after DM with regard to demographic profiles, lifestyle factors, the year of DM diagnosis, most comorbidities and medications (Table 1), except a modestly increased prevalence of chronic liver disease, gout, and malignancy among those without CKD. The SMDs of each variable between groups were lower than 0.1, suggesting that the distribution of each variable between groups was balanced (Table 1).

**Table 1:** Characteristics of patients with diabetes mellitus, with and without CKD after diabetes

	With CKD (n = 55,961)	Without CKD (n = 55,961)	p-value	SMDs
*Demographic profile*				
Age (years)	56.6 ± 13.6	56.5 ± 12.9	0.33	0.0058
Sex (Female %)	24,806 (44.3)	25,069 (44.8)	0.11	− 0.0209
*Lifestyle factors*				
Smoking (%)	359 (0.6)	343 (0.6)	0.545	0.0036
Alcoholism (%)	840 (1.5)	905 (1.6)	0.117	− 0.0094
*Years of DM diagnosis*			0.88	0.0092
2004	8450 (15.1)	8480 (15.2)		
2005	8547 (15.3)	8464 (15.1)		
2006	8049 (14.4)	8149 (14.6)		
2007	8156 (14.6)	8147 (14.6)		
2008	7790 (13.9)	7821 (14.0)		
2009	7765 (13.9)	7640 (13.7)		
2010	7204 (12.9)	7260 (13.0)		
*Comorbidity*				
Obesity (%)	1028 (1.8)	1017 (1.8)	0.806	0.0015
Hypertension (%)	32,865 (58.7)	32,773 (58.6)	0.577	0.0033
Hyperlipidemia (%)	26,045 (46.5)	26,192 (46.8)	0.379	− 0.0053
Chronic liver disease (%)	22,287 (39.8)	22,992 (41.1)	< 0.001	− 0.0257
COPD (%)	6648 (11.9)	6765 (12.1)	0.282	− 0.0064
Asthma (%)	5834 (10.4)	5909 (10.6)	0.464	− 0.0044
Parkinsonism (%)	436 (0.8)	425 (0.8)	0.707	0.0022
Gout (%)	15,892 (28.4)	16,286 (29.1)	0.009	− 0.0156
Malignancy (%)	4693 (8.4)	5423 (9.7)	< 0.001	− 0.0455
Mental disorders (%)	10,137 (18.1)	10,315 (18.4)	0.169	− 0.0082
Dementia (%)	603 (1.1)	632 (1.1)	0.407	− 0.0050
Proteinuria (%)	1373 (2.5)	1256 (2.2)	0.021	0.0138
Hypoglycemia events (%)	74 (0.1)	83 (0.2)	0.472	− 0.0043
*Relevant medications*				
ACEi (%)	17,386 (31.1)	17,485 (31.2)	0.523	− 0.0038
Allopurinol (%)	3172 (5.7)	3185 (5.7)	0.867	− 0.0010
Anti-depressants (%)	11,691 (20.9)	11,751 (21.0)	0.659	− 0.0026
Anti-psychotics (%)	15,026 (26.9)	15,279 (27.3)	0.089	− 0.0102
ARB (%)	23,734 (42.4)	23,401 (41.8)	0.044	0.0121
Aspirin (%)	18,118 (32.4)	18,061 (32.3)	0.716	0.0022
β-blockers (%)	25,470 (45.5)	25,412 (45.4)	0.728	0.0021
Benzodiazepine (%)	30,595 (54.7)	30,810 (55.1)	0.197	− 0.0077
Clopidogrel (%)	2302 (4.1)	2269 (4.1)	0.618	0.0030
COX-II inhibitor (%)	16,493 (29.5)	16,714 (29.9)	0.148	− 0.0086
Fibrate (%)	12,240 (21.9)	12,324 (22.0)	0.544	− 0.0036
NSAID (%)	51,571 (92.2)	51,559 (92.1)	0.894	0.0008
Statin (%)	25,411 (45.4)	25,441 (45.5)	0.857	− 0.0011
Warfarin (%)	725 (1.3)	715 (1.3)	0.791	0.0016
*Anti-diabetic agents*				
α-glucosidase inhibitor (%)	8556 (15.3)	8528 (15.2)	0.816	0.0014
Biguanide (%)	32,734 (58.5)	32,807 (58.6)	0.658	− 0.0026
DPP4 inhibitors (%)	5223 (9.3)	5199 (9.3)	0.805	0.0015
Insulin (%)	6555 (11.7)	6508 (11.6)	0.662	0.0026
Meglitinide (%)	6190 (11.1)	6041 (10.8)	0.153	0.0085
Sulfonylurea (%)	29,011 (51.8)	29,148 (52.1)	0.412	− 0.0049
Thiazolidinedione (%)	5872 (10.5)	5790 (10.4)	0.422	0.0048
*Duration of follow-up (years)*	4.3 ± 2.1	4.3 ± 2.1	0.10	
*Medians (IQR) (years)*	4.2 (2.5, 6.1)	4.3 (2.5, 6.1)		

*ACEi* angiotensin-converting enzyme inhibitor, *ARB* angiotensin receptor blocker, *CKD* chronic kidney disease, *COPD* chronic obstructive pulmonary disease, *DKD* diabetic kidney disease, *DM* diabetes mellitus, *DPP* dipeptidyl peptidase, *IQR* interquartile range, *NSAID* non-steroidal anti-inflammatory drug, *SMD* standardized mean difference

**Fig. 2:**
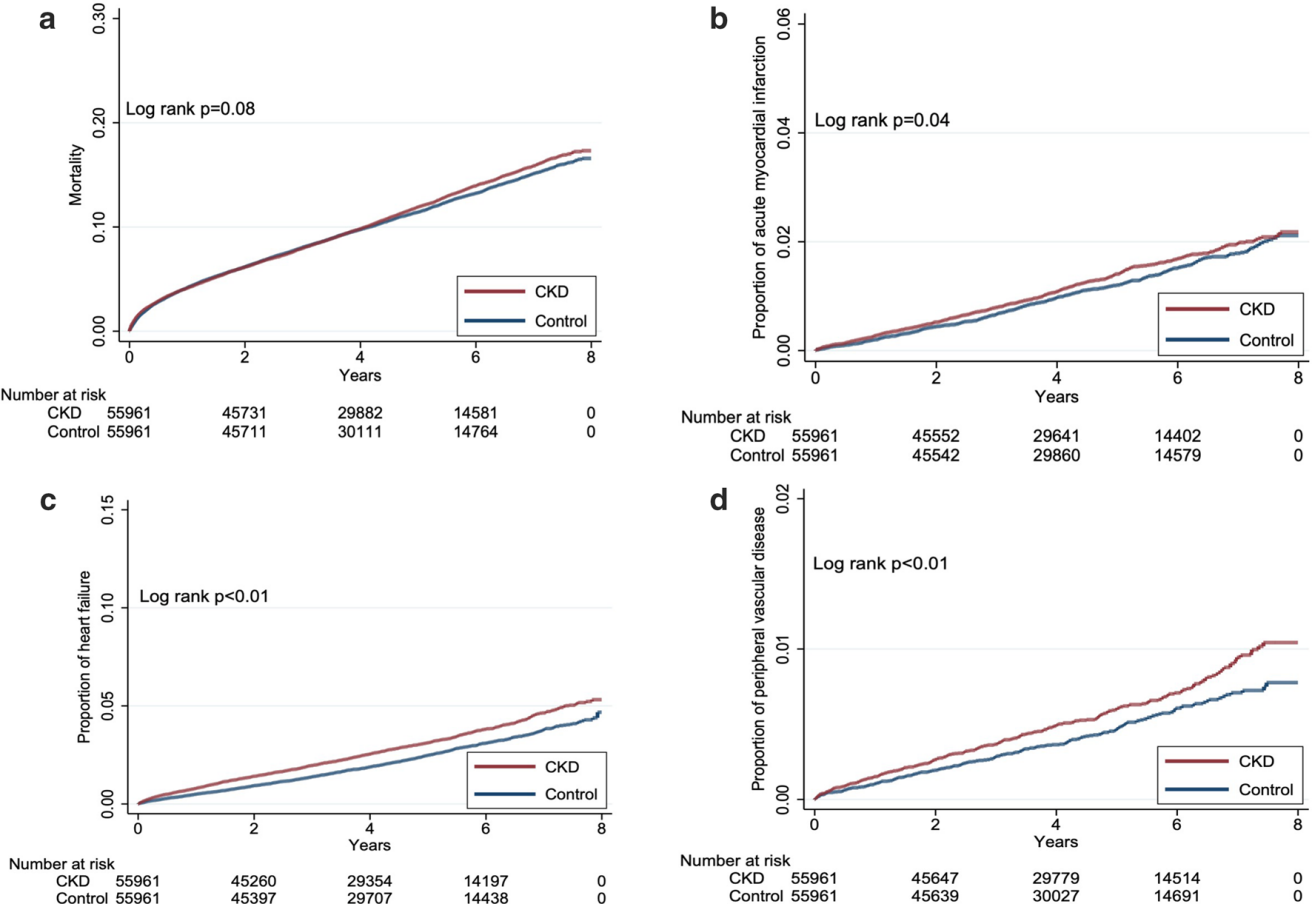
The Kaplan–Meier event curves for survival (**a**), incident heart failure (**b**), incident acute myocardial infarction (**c**), and incident peripheral vascular disease (**d**) among newly diagnosed diabetic patients in this study. *CKD* chronic kidney disease

**Fig. 3:**
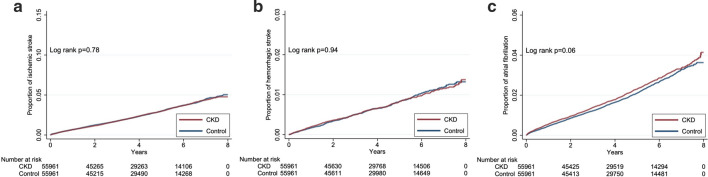
The Kaplan–Meier event curves for incident ischemic stroke (**a**), incident hemorrhagic stroke (**b**), and incident atrial fibrillation (**c**) among newly diagnosed diabetic patients in this study. *CKD* chronic kidney disease

After a median of 4.2 years of follow-up, 12,270 (11.0%) patients died, while 2,778 (2.5%), 1,250 (1.1%), 534 (0.5%), 2,914 (2.6%), 800 (0.7%), and 2,181 (1.9%) developed incident HF, AMI, PVD, IS, HS, and Afib, respectively (Table 2). Patients with incident DM and CKD afterward had at a significantly higher incidence of developing HF (*p* < 0.01), AMI (*p* = 0.04), and PVD (*p* < 0.01) than those without CKD (Fig. 2). Those with incident DM and CKD afterward had a trend of higher incidence of mortality (*p* = 0.08) and Afib (*p* = 0.06) than those without CKD, while no difference in the incidence of IS and HS was observed between those with and without CKD (Figs. 2 and 3). Cox proportional hazard regression showed that patients with incident DM and CKD afterward had a significantly higher risk of mortality (hazard ratio [HR] 1.1, 95% CI 1.06–1.14), developing HF (HR 1.282, 95% CI 1.19–1.38), AMI (HR 1.16, 95% CI 1.04–1.3), and PVD (HR 1.277, 95% CI 1.08–1.52) compared to those without CKD during follow-up, while the risk of IS, HS, and Afib did not differ between incident diabetic patients with and without CKD (Table 2). Sensitivity analyses based on a more restrictive set of CKD diagnostic codes showed that incident diabetic patients with CKD (n = 54,224) had a significantly higher risk of overall mortality (HR 1.098; 95% CI 1.06–1.14), HF (HR 1.282, 95% CI 1.19–1.38), AMI (HR 1.147, 95% CI 1.02–1.29), and PVD (HR 1.279, 95% CI 1.07–1.52) than those without (Table 3). In addition, another sensitivity analysis based on DKD-specific codes identified few patients with such diagnosis (n = 5,441). Cox proportional hazard regression showed that the CKD-associated risk of mortality (HR 1.218, 95% CI 1.08–1.38) and developing HF (HR 2.018, 95% CI 1.59–2.56) remained significant in this DKD-specific subcohort, while the risk of developing AMI (HR 1.28, 95% CI 0.88–1.85) and PVD (HR 1.401, 95% CI 0.82–2.40) was insignificant due to low event counts involving these endpoints (Additional file 1: Table S2).

**Table 2:** Risk of developing each cardiovascular morbidity according to the presence of CKD after DM or not

Outcomes	Events	Person-year	Incidence density*	Crude		Model A^&^	
				HR	95% CI	HR	95% CI
*Mortality*							
Matched control	6052	238,809.41	25.34	1	–	1	–
CKD after DM	6218	237,651.29	26.16	1.032	0.996–1.069	1.100	1.06–1.14^c^
*Heart failure*							
Matched control	1215	236,548.79	5.14	1	–	1	–
CKD after DM	1563	234,671.53	6.66	1.297	1.20–1.40^c^	1.282	1.19–1.38^c^
*Acute myocardial infarction*							
Matched control	591	237,555.71	2.49	1	–	1	–
CKD after DM	659	236,418.19	2.79	1.121	1.00–1.25^a^	1.160	1.04–1.30^a^
*Peripheral vascular disease*							
Matched control	234	238,301.22	0.98	1	–	1	–
CKD after DM	300	237,101.98	1.27	1.289	1.09–1.53^b^	1.277	1.08–1.52^b^
*Ischemic stroke*							
Matched control	1468	235,349.99	6.24	1	–	1	–
CKD after DM	1446	234,316.7	6.17	0.990	0.92–1.06	0.998	0.93–1.07
*Hemorrhagic stroke*							
Matched control	402	238,083.13	1.69	1	–	1	–
CKD after DM	398	237,019.87	1.68	0.995	0.87–1.14	0.967	0.84–1.11
*Atrial fibrillation*							
Matched control	1050	236,780.84	4.43	1	–	1	–
CKD after DM	1131	235,583.15	4.80	1.083	0.996–1.18	1.052	0.97–1.15

*CI* confidence interval, *CKD* chronic kidney disease, *DM* diabetes mellitus, *HR* hazard ratio

*per 1000 patient-year

^&^Incorporating age/gender, lifestyle factors, all comorbidities, and all medications

^a^*p* < *0.05*

^*b*^*p* < *0.01*

^*c*^*p* < *0.001*

**Table 3:** Risk of developing each cardiovascular morbidity according to the presence of CKD after DM or not, based on a restrictive CKD diagnostic code set (n = 54,224 per group)

Outcomes	Events	Person-year	Incidence density*	Crude		Model A^&^	
				HR	95% CI	HR	95% CI
*Mortality*							
Matched control	5853	231,470.74	25.29	1	–	1	–
CKD after DM	5985	230,546.27	25.96	1.026	0.99–1.06	1.098	1.06–1.14^c^
*Heart failure*							
Matched control	1179	229,256.82	5.14	1	–	1	–
CKD after DM	1523	227,644.35	6.69	1.301	1.21–1.40^c^	1.282	1.19–1.38^c^
*Acute myocardial infarction*							
Matched control	575	230,252.1	2.50	1	–	1	–
CKD after DM	637	229,347.34	2.78	1.112	0.99–1.25	1.147	1.02–1.29^a^
*Peripheral vascular disease*							
Matched control	227	230,979.43	0.98	1	–	1	–
CKD after DM	291	230,007.4	1.27	1.288	1.08–1.53^b^	1.279	1.07–1.52^b^
*Ischemic stroke*							
Matched control	1414	228,115.7	6.20	1	–	1	–
CKD after DM	1413	227,280.79	6.22	1.003	0.93–1.08	1.013	0.94–1.09
*Hemorrhagic stroke*							
Matched control	392	230,755.67	1.70	1	–	1	–
CKD after DM	385	229,933.54	1.67	0.986	0.86–1.14	0.958	0.83–1.10
*Atrial fibrillation*							
Matched control	1016	229,503.21	4.43	1	–	1	–
CKD after DM	1095	228,537.21	4.79	1.083	0.99–1.18	1.053	0.97–1.15

*CI* confidence interval, *CKD* chronic kidney disease, *DM* diabetes mellitus, *HR* hazard ratio

* per 1000 patient-year

^&^ Incorporating age/gender, lifestyle factors, all comorbidities, and all medications

^a^ *p* < *0.05*

^*b*^* p* < *0.01*

^*c*^* p* < *0.001*

**Fig. 4:**
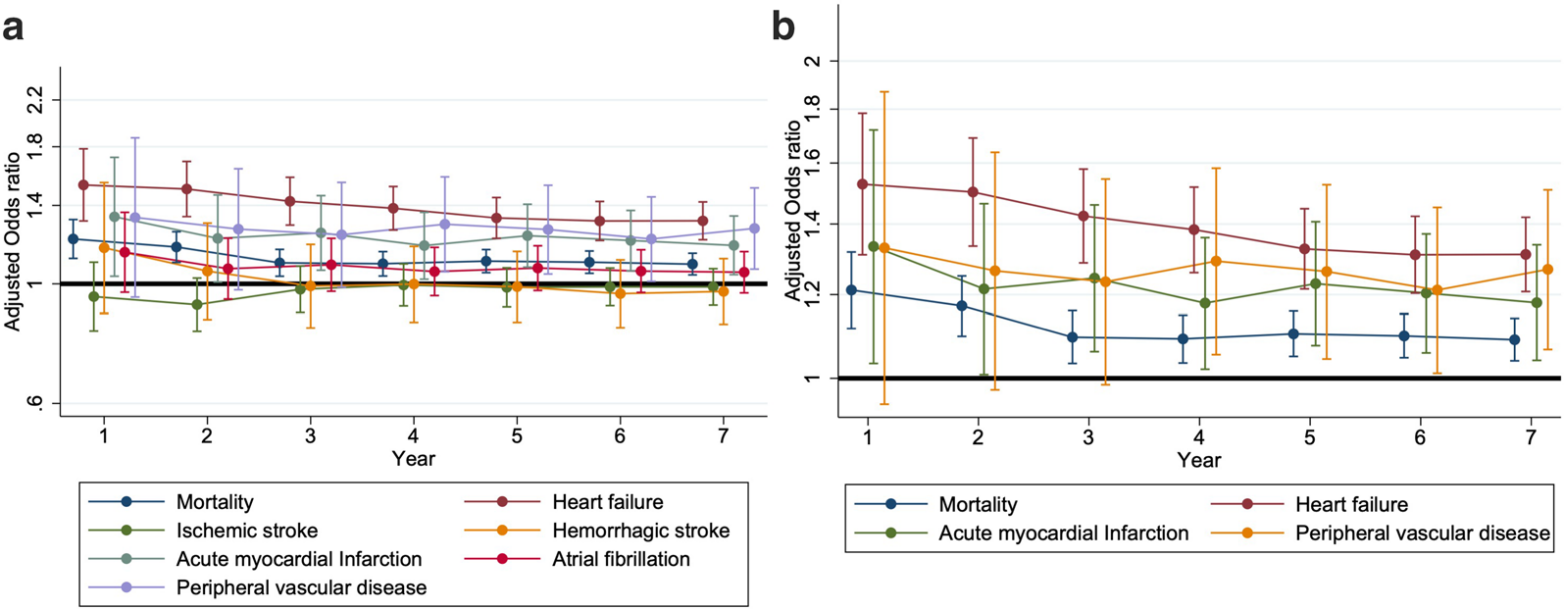
The adjusted odds ratio for developing different cardiovascular morbidities annually throughout the study period among participants

**Fig. 5:**
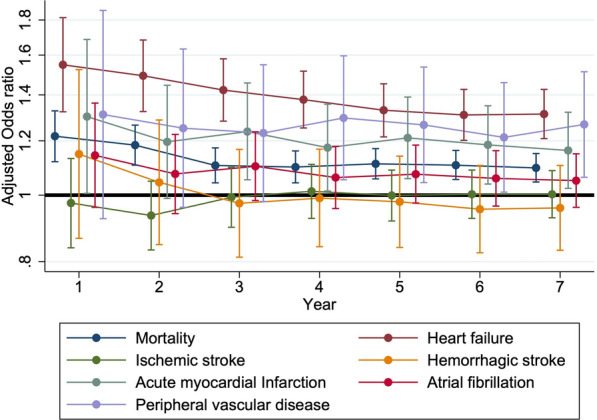
The adjusted odds ratio for developing different cardiovascular morbidities annually throughout the study period among participants identified based on a restrictive CKD diagnosis

We then examined the OR of mortality and that of developing each cardiovascular morbidity annually over 7 years of follow-up within the study period (Fig. 4a). Interestingly, the risk of each cardiovascular morbidity associated with CKD after incident DM followed different trajectories; we found that the CKD-associated risk of mortality and developing HF as well as AMI became significant soon after the diagnosis of DM was made, and remained significant throughout the study period (Fig. 4b). However, the risk of PVD conferred by CKD in patients with incident DM did not emerge until 4 years after DM was diagnosed (Fig. 4b). Finally, the CKD-associated risk of IS, HS and Afib in these patients was insignificant up to 7 years after the initial diagnosis of DM (Fig. 4a). Using repeated measures of analysis of variance (ANOVA), we showed that statistical differences existed in the CKD-associated annual risk of incident HF (*p* < 0.001) and AMI (*p* = 0.016), with a temporal trend of gradual decline since the initial diagnosis of DM (Fig. 4b). Similar temporal trends of the CKD-associated risk related to the development of different cardiovascular morbidities are shown in the restrictive CKD cohort (Fig. 5).

## Discussion

In this study, we used a population-based cohort with a propensity score-matching strategy to investigate the influences of incident CKD on the risk of multiple cardiovascular morbidities among patients with newly diagnosed DM. After accounting for other cardiovascular risk and protective factors such as demographic profiles, comorbidities, and medications, we found that the CKD-associated risk of mortality, HF, AMI, and PVD was significant during follow-up, while the risk of IS, HS, and Afib was not. Interestingly, the risk associated with each cardiovascular morbidities differed over time; for mortality, HF, and AMI, the risk remained significant for 7 years consecutively while the risk for PVD did not emerge until 4 years after DM diagnosis. A temporal trend of slight risk decline was also noted. These findings are expected to facilitate our planning of an optimal complication detection and management strategy for those with a fresh diagnosis of DM.

Studies have consistently showed that the incidence of cardiovascular complications in patients with DM improved during the recent decade compared to that in 1990s [7, 29]. This improvement likely stems from the advances in primary care delivery, an increased understanding of the importance of lifestyle modifications, and the pharmacological therapeutics for secondary cardiovascular prevention [30]. To further lower the risk of diabetic cardiovascular complications, we need to gain more insight into potential modifiers of such risk, one of which is CKD. An intricate and bi-directional link between renal health and cardiovascular diseases has been recognized, with the concept of cardiorenal syndrome introduced one decade ago [31]. The presence of DM contributes to the constant propagation of this vicious cycle through inducing micro- and macro-vascular injuries, increasing advanced glycation endproducts (AGEs), and the accompanying comorbidities such as dyslipidemia [32]. However, for most practitioners, the detection of CKD in patients with DM raises the concern of renal progression and the risk of ESRD, while other adverse influences of CKD in these patients can be under-appreciated. Indeed, the occurrence of CKD after DM not only leads to ESRD, but also increases the risk of cardiovascular diseases including coronary artery disease, AMI, and HF in patients with DM [33]. Apart from the co-existence of traditional cardiovascular risk factors, CKD also brings forth non-traditional cardiovascular risk factors such as divalent ion imbalance, pro-atherogenic uremic toxins, vascular calcification, and chronic inflammation, all of which set the background for the accelerated development of cardiovascular disorders [34, 35]. Thus, it is not unexpected that we identify a close relationship between CKD and incident HF, AMI, and PVD in these diabetic patients (Table 2). However, the risk seems to decrease slightly after the initial DM diagnosis (Fig. 4b). We presume that this may result from the harm-reduction treatments administered for CKD after patients received the diagnosis of DM, such as renin–angiotensin–aldosterone system blockade, whose adverse effects during earlier CKD are less prominent.

We also found that the presence of CKD did not significantly increase the risk of IS, HS, and Afib in patients with newly diagnosed DM. Several reasons may be responsible for this finding. First, it is plausible that in these patients with DM of a short duration, they had relatively earlier stages of CKD; early CKD increases the risk of ischemic stroke through its concurrence with other predisposing factors such as hypertension, most of which were already adjusted for in our analyses. Similar findings are reported recently; Cabrera et al. [36], using clinical practice data from United Kingdom, showed that CKD did not alter the risk of IS among a large cohort of patients with DM. Oh and colleagues also demonstrated that there was no association between lower estimated glomerular filtrate rate (eGFR) levels and the risk of HS in a population-based cohort of Korean patients [37]. Prior studies concluded that the risk of stroke, especially HS, differed depending on ethnicity, while Asians were particularly at risk [38]. It is thus possible that the risk differences in IS and HS introduced by CKD are diminished in our diabetic participants due to their higher risk at baseline. Thirdly, there are arguments that in those with early CKD, the severity of reduced GFR or proteinuria is more akin to a surrogate for the severity of underlying cardiovascular disease instead of exhibiting a causal relationship [39]. Finally, another possibility would be that the CKD-associated risk for IS, HS, and Afib takes a relatively longer time to emerge, and our patients just had newly diagnosed DM. More studies are still needed to affirm our findings and elucidate the potential mechanisms.

We showed that the CKD-associated risk of PVD did not emerge until 4 years after the initial diagnosis of DM (Fig. 4b). This finding is echoed by those from prior reports, which described that the risk of PVD was closely associated with a longer duration of DM and an obvious increase in PVD prevalence was not observed until middle-age or higher [40]. Another study comparing the comorbidity profiles between patients with pre-diabetes and established DM showed that the prevalence of PVD was significantly lower among the former than the latter [41], supporting the existence of a duration-dependent risk of PVD in diabetic patients. Impairment of vasculature in diabetes, from micro- to macro-vascular diseases, results from endothelial and vascular smooth muscle cell dysfunction related to the increased oxidative stress, AGE accumulation, a perturbed anti-oxidant system, surging inflammatory mediators, etc. [42]. This can be further exacerbated by the adverse influences posed by CKD-induced uremic milieu, epigenetic changes associated with calcium/phosphate imbalance [43], vitamin D deficiency [44], and others, culminating in the development of vasculopathy such as vascular calcification involving limb arteries. However, the risk of macrovascular complications associated with DM might require time to develop, even when it is compounded by incident CKD. From this perspective, prioritization of complication detection and management may be considered depending on the specific type of cardiovascular diseases in patients with DM.

Major advances in the management of DM and its cardiovascular complications have been discovered recently, including pharmacological innovations such as sodium glucose cotransporter 2-inhibitors, glucagon-like peptide 1 receptor agonists, and risk factor amelioration including home blood pressure monitoring and endothelial dysfunction reduction [45–49]. In addition, it has been reported that anti-diabetic medications may differ in their effects on cardiovascular risk profile among patients with DM [50, 51]. It is likely that the phenomenon we identified in this study may be altered in the near future, and more contemporary study may be needed to elucidate this issue.

Our study has its strengths and limitations. The diabetic cohort we used, LCDP, was well maintained and contained an extensive array of clinical variables recorded; the large sample size in this study attenuated the possibility of imbalances between groups. This strategy was further supplemented by the propensity score-matched design, with credible results obtained. Nonetheless, several limitations exist. First, our study was a retrospective analysis of prospectively collected data, and the diagnosis of cardiovascular outcomes was made based on physicians’ discretion. In addition, the severity of each cardiovascular morbidity was unavailable in this database, precluding relevant analyses. The applicability of exclusion criteria might lead to preferential selection, although the proportion of excluded patients from LCDP was small. We did not validate the accuracy of the diagnostic code combinations for each comorbidity in the LCDP database, but results from several prior reports were in support of their validity [23, 24, 26–28]. Besides, the causes of death were unavailable in this study, precluding a detailed analysis. We were unable to analyze the subgroup of patients with advanced CKD, since we specifically searched for those with newly diagnosed DM, and a very low proportion of them had severe CKD. Finally, our cohort consisted uniformly of diabetic patients of Asian ethnicity, and whether our findings could be extrapolated to those of other ethnicities remain undetermined. Studies incorporating patients from different areas are needed to affirm and extend our findings.

## Conclusion

Using a population-based cohort of patients with newly diagnosed diabetes, we examined whether the CKD-associated risk of developing cardiovascular diseases differed depending on the disease type and the duration of DM. We were able to show that the risk profile could be divergent; the risk of mortality, HF, and AMI introduced by CKD remained significant throughout the follow-up period, while the risk of PVD did not emerge until 4 years after the initial DM diagnosis. On the other hand, among these newly diagnosed DM patients, the CKD-associated risk of IS, HS, and Afib was insignificant. These findings are expected to shed light on the optimal strategy for detecting early cardiovascular complications among patients with incident DM, and facilitate the timely administration of cardiovascular care.

## Supplementary Information

**Additional file 1: Table S1.** Diagnostic and procedure codes for identifying cardiovascular morbidities in this study. **Table S2.** Risk of developing each cardiovascular morbidity according to the presence of DKD or not, based on a specific diagnostic codes for DKD (n = 5441 per group).

## Data Availability

The raw data for conducting this analysis are unavailable due to administrative regulations.

## Abbreviations

Afib: Atrial fibrillation
AGE: Advanced glycation endproduct
AMI: Acute myocardial infarction
ANOVA: Analysis of variance
CI: Confidence interval
CKD: Chronic kidney disease
DKD: Diabetic kidney disease
DM: Diabetes mellitus
eGFR: Estimated glomerular filtration rate
ESRD: End-stage renal disease
HF: Heart failure
HR: Hazard ratio
HS: Hemorrhagic stroke
IS: Ischemic stroke
KDIGO: Kidney Disease Improving Global Outcomes
LCDP: Longitudinal Cohort of Diabetes Patients
NHIRD: National Health Insurance Research Database
OR: Odds ratio
PVD: Peripheral vascular disease
SMD: Standardized mean differences

## Code availability

Custom codes were used in this study.
