# Supplementary material for: The risk trajectory of different cardiovascular morbidities associated with chronic kidney disease among patients with newly diagnosed diabetes mellitus: a propensity score-matched cohort analysis
Source: Cardiovasc Diabetol. 2021 Apr 24;20:86. doi: 10.1186/s12933-021-01279-6 (PMC8070330; doi:10.1186/s12933-021-01279-6)
Supplement: Supplementary file 1 — Additional file 1: Table S1. Diagnostic and procedure codes for identifying cardiovascular morbidities in this study. Table S2. Risk of developing each cardiovascular morbidity according to the presence of DKD or not, based on a specific diagnostic codes for DKD (n = 5441 per group). [file 12933_2021_1279_MOESM1_ESM.doc]

**Table S1**. Diagnostic and procedure codes for identifying cardiovascular morbidities in this study

| **Heart failure** | **Acute myocardial infarction** | **Peripheral vascular disease** | **Ischemic stroke** | **Haemorrhagic stroke** | **Atrial fibrillation** |
| --- | --- | --- | --- | --- | --- |
| 428.x | 410.x | 441.x | 433.x | 430.x | 427.x |
| 402.01 |  | 443.9 | 434.x | 431.x |  |
| 402.11 |  | 785.4 | 436.x | 432.x |  |
| 402.91 |  | V43.4 |  |  |  |
| 404.01 |  | Procedure code 3848 |  |  |  |
| 404.03 |  |  |  |  |  |
| 404.11 |  |  |  |  |  |
| 404.13 |  |  |  |  |  |
| 404.91 |  |  |  |  |  |
| 404.93 |  |  |  |  |  |

**Table S2.** Risk of developing each cardiovascular morbidity according to the presence of DKD or not, based on a specific diagnostic codes for DKD (n=5,441 per group)

| **Outcomes** | **Events** | **Person-year** | **Incidence density*** | **Crude** | | **Model A^&^** | |
| --- | --- | --- | --- | --- | --- | --- | --- |
|  |  |  |  | **HR** | **95% CI** | **HR** | **95% CI** |
| *Mortality* |  |  |  |  |  |  |  |
| Matched control | 522 | 23,532.13 | 22.18 | 1 | - | 1 | - |
| DKD | 595 | 23,937.76 | 24.86 | 1.123 | 0.999 – 1.26 | 1.218 | 1.08 – 1.38^b^ |
| *Heart failure* | |  |  |  |  |  |  |
| Matched control | 115 | 23,314.67 | 4.93 | 1 | - | 1 | - |
| DKD | 230 | 23,463.4 | 9.80 | 1.985 | 1.59 – 2.48^a^ | 2.018 | 1.59 – 2.56^a^ |
| *Acute myocardial infarction* | |  |  |  |  |  |  |
| Matched control | 63 | 23,395.91 | 2.69 | 1 | - | 1 | - |
| DKD | 79 | 23,792.96 | 3.32 | 1.226 | 0.88 – 1.71 | 1.280 | 0.88 – 1.85 |
| *Peripheral vascular disease* | |  |  |  |  |  |  |
| Matched control | 26 | 23,486.28 | 1.11 | 1 | - | 1 | - |
| DKD | 37 | 23,849.56 | 1.55 | 1.402 | 0.85 – 2.32 | 1.401 | 0.82 – 2.40 |
| *Ischemic stroke* | |  |  |  |  |  |  |
| Matched control | 143 | 23,176.43 | 6.17 | 1 | - | 1 | - |
| DKD | 165 | 23,536.79 | 7.01 | 1.132 | 0.91 – 1.42 | 1.199 | 0.95 – 1.52 |
| *Hemorrhagic stroke* | | |  |  |  |  |  |
| Matched control | 41 | 23,446.9 | 1.75 | 1 | - | 1 | - |
| DKD | 44 | 23,868.23 | 1.84 | 1.057 | 0.69 – 1.62 | 1.002 | 0.64 – 1.57 |
| *Atrial fibrillation* |  |  |  |  |  |  |  |
| Matched control | 108 | 23,326.01 | 4.63 | 1 | - | 1 | - |
| DKD | 105 | 23,729.63 | 4.42 | 0.954 | 0.73 – 1.25 | 1.081 | 0.82 – 1.43 |

*DKD, diabetic kidney disease; DM, diabetes mellitus; HR, hazard ratio*

^*^ per 1000 patient-year

^&^ Incorporating age/gender, lifestyle factors, all comorbidities, and all medications

^a^ *p < 0.001*

*^b^ p < 0.01*
